# Immunosenescence: Aging and Immune System Decline

**DOI:** 10.3390/vaccines12121314

**Published:** 2024-11-23

**Authors:** Priyanka Goyani, Rafail Christodoulou, Evros Vassiliou

**Affiliations:** 1 Department of Biological Sciences, Kean University, Union, NJ 07083, USA; goyanip@kean.edu; 2 Department of Radiology, School of Medicine, University of Patras, 265 04 Rio, Greece; up1063080@ac.upatras.gr

**Keywords:** cytokines, inflammation, immunoscenence, thymus, immune system

## Abstract

Immunosenescence, a systematic reduction in the immune system connected with age, profoundly affects the health and well-being of elderly individuals. This review outlines the hallmark features of immunosenescence, including thymic involution, inflammaging, cellular metabolic adaptations, and hematopoietic changes, and their impact on immune cells such as macrophages, neutrophils, T cells, dendritic cells, B cells, and natural killer (NK) cells. Thymic involution impairs the immune system’s capacity to react to novel antigens by reducing thymopoiesis and shifting toward memory T cells. Inflammaging, characterized by chronic systemic inflammation, further impairs immune function. Cellular metabolic adaptations and hematopoietic changes alter immune cell function, contributing to a diminished immune response. Developing ways to reduce immunosenescence and enhance immunological function in the elderly population requires an understanding of these mechanisms.

## 1. Introduction

**Figure 1:**
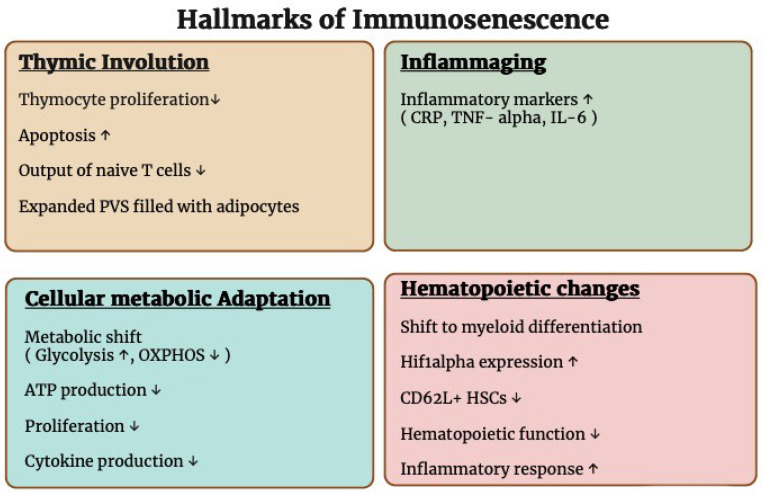
Hallmarks of immunosenescence. The primary characteristics of immunosenescence, including thymic involution, inflammaging, cellular metabolic adaptation, and hematopoietic changes. Each section highlights the physiological and molecular changes that contribute to reduced immune competence and an increased inflammatory response with aging. Arrows indicate the direction of change: ↑ denotes an increase and ↓ denotes a decrease.

Immunosenescence is an age-related decline in immune system function that makes people susceptible to autoimmune disorders, cancer, and infections. As the global population ages, understanding immunosenescence is becoming increasingly important for public health. The immune system’s gradual deterioration is a byproduct of complex interactions between intrinsic cellular aging changes and extrinsic factors (see Figure 1).

Aging impacts adaptive and innate immunity. The innate immune system shows decreased efficacy in pathogen recognition and clearance. Adaptive immunity, responsible for targeted responses, suffers from reduced production and function of T and B lymphocytes. These changes collectively reduce the body’s ability to build up effective immune responses, making elderly individuals more susceptible to diseases.

## 2. Hallmarks of Immunosenescence

### 2.1. Thymic Involution

Thymic involution is characterized by two processes, a reduction in thymocyte proliferation and a increase in apoptosis in the thymic cortex, which leads to a decreased output of naïve T cells [1]. A study by Wu et al. showed that medullary thymic epithelial cells (mTECs) in the thymus of aged mice exhibit features of SASP (senescence-associated secretory phenotype), which may be responsible for the inflammatory environment and impaired thymopoiesis observed during thymic involution [2]. A study by Reis et al. showed that the expression of the forkhead box N1 (FOXN1) transcription factor declined with age, which is crucial for epithelial cell development of the thymus [3]. Thymocyte development and selection are regulated by FOXN1, and the loss of FOXN1 leads to a decline in thymopoiesis. With aging, the thymic perivascular space (PVS) enlarges and is filled with fat cells, which further interrupt thymopoiesis [4]. These alterations result in a decline in the thymic function, which, in turn, reduces the thymus’s capacity to generate new T cells, causing the immune system to become more reliant on memory T cells [5].

The reduced interleukin-7 (IL-7) cytokine expression with age hinders the development of thymocytes and proliferation [6]. However, the molecules of gap junction Connexin 43 expression do not decline with age, which suggests that thymic epithelial cells (TECs) are replaced by cells and they do not have the ability to produce IL-7. As a result, elderly people’s immune system becomes more susceptible to infections, cancer, and auto-immune diseases.

The process of involution also involves significant structural thymic changes, including a reduction in size, a decrease in functional thymic tissue, and fatty replacement of the thymic parenchyma, which has been confirmed by various imaging modalities [7]. These changes can be visualized clearly and confirm immunosenescence in aging. Computed Tomography (CT) is one of the most commonly used tools to assess thymic changes in aging. In young adults, the thymus is typically visualized as a triangular soft tissue structure with homogeneous attenuation. With aging, the thymus undergoes fatty replacement which can appear on CT as a gradual decrease in thymic tissue attenuation [8,9]. Since most of the thymus is replaced by fat, residual thymic tissue appears as small tissue islands of soft tissue within fat. Magnetic Resonance Imaging (MRI) provides more detailed characterization, making it ideal for visualizing thymus involution. On T1-weighted images, the thymus is intermediate in intensity, similar to soft tissue. With age and as fat replaces the soft tissue, it begins to have high signal intensity that is consistent with fatty replacement [10]. Similarly, T2-weighted images show low-to-intermediate signal intensity in younger individuals. As fatty replacement occurs, there is an increase in signal density, although it is less conspicuous compared to T1W images [11]. Positron Emission Tomography (PET) scans with the use of FDG provide insight into the metabolic activity of the thymus during immunosenescence. Normal aging thymus involution exhibits low FDG uptake that indicates reduced metabolic activity as it is predominantly replaced by fat (reviewed by Ferdinand et al.) [12]. In contrast to aging, thymic rebound hyperplasia, which is common after several months of immunosuppression caused by steroid or chemotherapy, has higher uptake of the tracer.

Although X-rays and ultrasound, in theory, can be used to assess thymic involution, they are more commonly used in younger individuals, since they have several diagnostic limitations in the context of thymic involution. As the fatty replacement commences with age, it often becomes indistinguishable on X-rays, making it difficult to capture subtle changes. Ultrasound faces its own challenges, reducing its sensitivity for detecting involution. Overall, X-rays and ultrasound provide limited diagnostic value in the assessment of thymic involution during immunosenescence. CT and MRI offer superior imaging capabilities that capture the structural changes with detail, while PET adds the functional component to the equation. In combination, the various imaging modalities are valuable tools for confirming the diagnosis of thymic involution in the setting of immunosenescence and setting the condition apart from other thymic pathologies.

### 2.2. Inflammaging

Inflammaging is a gradual increase in chronic inflammation that occurs with aging, characterized by higher concentrations of inflammatory markers in the body, including tumor necrosis factor-alpha (TNF-α), C-reactive proteins (CRP), and interleukins such as IL-6, IL-1 receptor antagonist (IL-1ra), IL-18, and IL-1b [13,14,15,16,17]. Older adults with elevated IL-6 levels and TNF-α in their blood are more likely to have less muscle mass and weaker muscles [18]. Age-related chronic inflammation is not directly caused by infection. This is driven by cell damage from free radicals, an imbalance in inflammatory cytokines, and cellular senescence. Aging leads to increased production of p16 (a tumor suppressor protein) and sterile α-motif domain- and HD domain-containing protein 1 (SAMHD1) in mouse models [19]. Additionally, aging alters the gut microbiota, resulting in increased lipopolysaccharide (LPS) production, which activates nuclear factor-kappa B (NF-κB), suggesting that gut bacteria’s LPS contributes to age related inflammation. NF-κB activation increases the expression of IL-6, which may lead to an increase in inflammation [20]. It is believed that these inflammatory markers affect an adult’s capacity to fight off infection, which leads to a decrease in overall immune function.

Chronic inflammation plays a significant role in the development and progression of cardiovascular diseases (CVDs). Elevated levels of inflammatory markers, such as high-sensitivity C-reactive protein (hsCRP), have been associated with an increased risk of cardiovascular events [21].

### 2.3. Cellular Metabolic Adaptations

Senescent T cells exhibit metabolic reprogramming characterized by a transition from oxidative phosphorylation to glycolysis [22]. This age-related adaptation results in less efficient ATP production, compromising cellular processes essential for immune function, including proliferation and cytokine production. Consequently, T cells exhibit a reduced capacity for proliferation and cytokine production, crucial for a robust immune response.

Aging significantly impacts mitochondrial DNA (mtDNA) quality and quantity [23,24]. Specifically, mtDNA number decreases with age, leading to reduced mitochondrial biogenesis and impaired energy production capacity. This decrease in mtDNA number compromises cell function, as mitochondria play a crucial role in powering cellular processes. Senescent T cells shift their energy production towards glycolysis for metabolic needs. Inhibiting p38 MAPK signaling can promote mitochondrial biogenesis and autophagy [25]. This could suggest a potential therapeutic strategy to rejuvenate aged immune cells, thereby enhancing overall immune capabilities.

### 2.4. Hematopoietic Changes

The aging process profoundly impacts the hematopoietic system, particularly hematopoietic stem cells (HSCs) [26]. Hematopoietic stem cells (HSCs) undergo a significant shift towards myeloid differentiation, altering the composition and functionality of immune cells [27,28,29,30]. This shift is driven by increased expression of hypoxia-inducible factor 1-alpha, leading to inflammatory responses. Additionally, aging impairs HSCs’ diversity and differentiation potential, resulting in reduced polymorphism and a decreased number of CD62L+ HSCs, which are crucial for lymphocyte homing in secondary lymphatic organs. Aged HSCs show general hypermethylation [31]. Specific transcription factors (TFs) contribute to myeloid bias associated with aging [32]. Knocking down Klf5 in aged long-term HSCs enhances lymphoid production and reduces myeloid output. In contrast, the knocking down of Ikzf1 in young LT-HSCs diminishes lymphoid production. These hematopoietic alterations contribute to the general decline in immune function seen in aging.

## 3. Impact of Aging on Immune Cells

### 3.1. Macrophages

**Figure 2:**
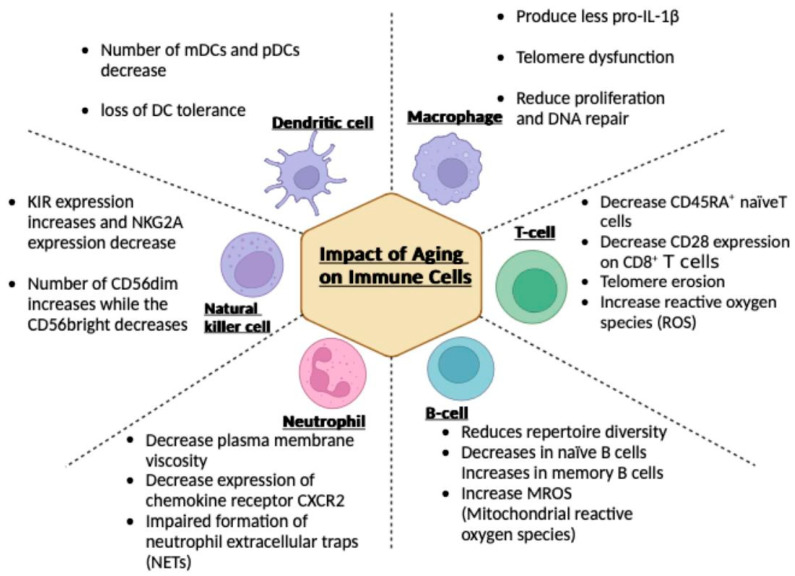
Impact of Aging on Immune Cells. The effects of aging on various immune cell types, including dendritic cells, macrophages, T cells, B cells, natural killer cells, and neutrophils. Key changes involve reduced cell numbers, altered expression markers, decreased proliferation, telomere dysfunction, and increased reactive oxygen species (ROS), indicating a decline in immune function with age.

Aging imparts a variety of changes to the various immune cells (see Figure 2). Older mice macrophages produced less pro-IL-1β than macrophages from young mice [33]. The macrophages’ capacity to control inflammation and fight infection is hampered by this lack of cytokine synthesis. Age-related dysregulation of genes linked to interferon-gamma (IFN-γ) responses has been observed in old mice [34]. IFN-γ is a crucial cytokine for immune control and tissue repair. A study by Zhang et al. [34] showed decreased inflammatory responses during muscle regeneration after acute injury, hindering efficient healing.

Telomere loss contributes to oxidative stress, abnormal macrophage mitochondria, and hyperactivation of the NLRP3 inflammasome [35]. Research by Dube et al. demonstrated that older animals’ macrophages express more pro-inflammatory genes and have reduced capabilities for cellular proliferation and DNA repair [36]. These alterations impair the wound healing mechanism, which causes older mice to experience increased tissue damage and a delayed resolution of inflammation. Changes in macrophages as a result of aging, emphasize a broader damage to the immune system. and its contribution to age-related consequences from acute injuries, which, in older people, can develop into chronic ones.

### 3.2. T Cells

As people age, CD8^+^ T cells and CD45RA^+^ naïve T cells decrease, but CD4^+^ T cells do not change [37]. This alteration reduces the immune system’s response to novel antigens, as opposed to it being highly responsive. Aged CD4^+^ and CD8^+^ naïve T cells exhibit elevated immune suppression and checkpoint regulation gene expression [38]. Additionally, CD28 expression decreases on CD8^+^ T cells, which is vital for the survival of T cells and their activation [39]. T cell receptor repertoire attrition reduces diversity, especially in CD8^+^ T cells [40]. This suggests that T cells have changed functional capacities, which increase susceptibility to infections and lower the effectiveness of vaccinations in older persons.

Furthermore, a history of multiple cell division, short telomeres, and a general lack of ability to proliferate are all indicated by high expression of CD57 on CD8^+^ T cells [41]. In peripheral blood, CD8^+^ T cells from healthy donors exhibit higher p53β expression and lower Δ133p53, indicating senescent cell accumulation (CD28^−^ CD57^+^) that is age-dependent [42]. Within human T cells, the endogenous p53 isoforms p53β and Δ133p53 function as physiological regulators of senescence and proliferation. Elevated p53β expression may increase autoimmune diseases risk, contributing to chronic inflammation.

Telomere erosion is caused by prolonged stimulation throughout the course of a person’s life due to T cells, with high proliferation propensity upon activation [43]. Upon activation, highly differentiated CD8^+^CD28^−^CD27^−^ T cells progress toward a replicative end stage, as indicated by telomerase down-regulation [44,45]. A strategy to improve the function of T cells in the elderly could involve increasing telomerase activity, as telomerase expression gradually decreases during T cell differentiation [45]. Oxidative stress and significant protein oxidative modification result from a rise in reactive oxygen species and a decrease in proteasome activity, respectively [46]. These events set the stage for cellular senescence and inflammation.

### 3.3. B Cells

Aging reduces the diversity of the B cell repertoire by influencing the selection process during B cell affinity maturation [47]. Decreases in naïve B cells and increases in memory B cells are two age-related changes in B cell populations. The immune system’s capacity to react to novel antigens is compromised by this change. Certain aged B cell subsets show elevated mitochondrial mass and mitochondrial reactive oxygen species because of age-related changes to B cell mitochondrial functioning [48]. Reduced mitochondrial energy production and abnormalities in one-carbon metabolism, which are necessary for amino acid and nucleotide synthesis and the antibody production and activation of B cells, are the outcomes of these alterations.

Lee et al. reported that compared to B cells from younger individuals, older individuals’ B cells do not exhibit fundamental abnormalities in their ability to proliferate and develop into antibody-secreting cells in vitro [49]. The GC response is delayed by age-related intrinsic B cell alterations, but this is not the cause of the compromised antibody-secreting response.

Rodriguez-Zhurbenko et al. found that as people aged, their B-1 cell percentage and their capacity to secrete IgM spontaneously declined [50]. Additionally, healthy donors over 65 years of age had significantly lower levels of XBP-1 and Blimp-1 transcription factor expression than did healthy young donors. However, PAX-5, a hallmark of non-secreting B cells, was much greater. Rodriguez-Zhurbenko et al. also found variation in the utilization of VH- and DH-specific genes and a decreased IgM antibody repertoire diversity in B-1 cells derived from elderly donors compared to younger ones. When combined, these modifications may result in decreased antibody production and a diminished capacity to react to immunization and novel antigens.

### 3.4. Neutrophils

Age-related increases in infection rates may result from structural alterations in neutrophils, such as lower plasma membrane viscosity and diminished adhesion capabilities [51]. When compared to neutrophils that are not from elderly people, senescent neutrophils have increased phagocytic activity [52,53]. Although this may appear to be a good thing, if neutrophils are few where they are needed, the immune system may be affected. According to a Study by Weisel et al., the expression of the chemokine receptor CXCR2, which is essential for directing neutrophils to the inflammatory location, is reduced [54]. Because of their decreased expression of CXCR2, elderly neutrophils are less likely to be drawn to the site of inflammation, which may influence the inflammatory response.

Neutrophils’ capacity to travel to and infiltrate infection sites is impacted by changes in neutrophil trafficking [55]. A reduced ability to eliminate pathogenic *E. coli* during septic peritonitis was noted in elderly mice, with increased neutrophil recruitment during LPS-induced peritonitis but not aseptic peritonitis [56]. Older mice’s neutrophils showed decreased *E. coli* killing and decreased reactive oxygen species (ROS) production after LPS priming. An important loss of neutrophil activity that leads to a weakened immune response is shown by the elderly population’s impaired ability to generate neutrophil extracellular traps (NETs) [57].

### 3.5. Natural Killer (NK) Cells

As people age, killer immunoglobulin-like receptor (KIR) expression increases, and NKG2A expression decreases [58,59]. NKG2A serves as an inhibitory receptor; inhibitory signaling may be diminished because of decreased NKG2A expression. This could result in increased lysis of healthy cells, which triggers autoimmunity. Isolated NK cells from elderly people produce more IL-8 responses to cytokine stimulation [60]. However, compared to younger NK cells, the amount of IL-8 produced is noticeably smaller.

Elderly patients exhibit alterations in the distribution of NK cell subsets instead of a simple drop in cell numbers, in contrast to adult patients, who show a decline in the overall NK cell count [61]. It has been demonstrated that as people age, the CD56dim subset increases, while the CD56bright subset decreases [62,63]. Aging impairs NK cells’ proliferative capacity [64]. In response to immunological challenges, younger individuals (<41 years) demonstrate robust NK cell proliferation, whereas elderly individuals (41–80 years) show reduced proliferation capacity.

### 3.6. Dendritic Cells

Dendritic cell (DC) development, cytokine production, and antigen presentation are all hampered by aging and are essential for monitoring and controlling immune responses [65]. Blood samples from aged patients showed lower myeloid DC (mDCs) counts than those from younger subjects [66]. When the antigen-presenting ability of elderly and young plasmacytoid DCs (pDCs) was evaluated, it was shown that the older patients’ pDCs were less able to stimulate IFN-γ secretion and CD4^+^ and CD8^+^ T cell proliferation [67]. Jing et al. reported a noteworthy decline in pDC counts with aging [68]. Older adults’ weakened immune response to viral infections is a result of age-related alterations in pDCs.

Research by Bashir et al. has illuminated how aging affects gut dysbiosis and how this affects the loss of DC tolerance [69]. For instance, DCs produced in young mice with gut dysbiosis or in elderly mice show a notable decrease in immunological tolerance. Compared to DCs from young, healthy mice, they are unable to moderate the overactivation of CD4^+^ T cells and to successfully stimulate the production of Tregs. The general immunosenescence that older adults experience is influenced by the reported loss in these processes, which results in a weakened immune system.

## 4. Comprehensive Approaches for Managing Age-Related Immune Decline

**Figure 3:**
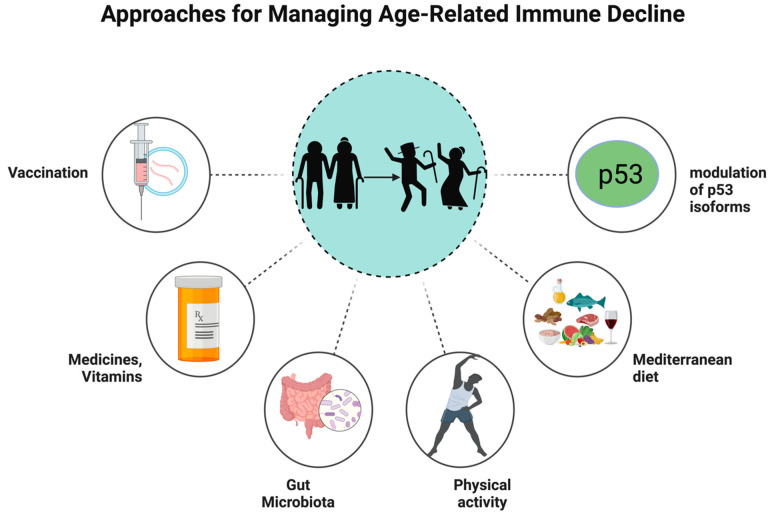
Approaches for managing age-related immune decline. Vaccination, p53 modulation, a Mediterranean diet, exercise, gut microbiome support, and supplements are promising strategies to combat immunological aging. They aim to lower inflammation and strengthen older people’s immune systems.

To change the immune landscape caused by aging in seniors, a variety of approaches seem promising (see Figure 3). New vaccine strategies are one of several strategies that warrant further exploration. The immune system’s response is impacted by modifications to hematopoietic stem cells, especially a move towards a myeloid lineage [27]. Elderly people who receive high-dose influenza vaccines produce more antibodies and have better protection against the influenza virus [70]. Pneumococcal 23-valent and 13-valent offer protection to elderly people against usual Streptococcus pneumoniae serotypes containing several antigen subtypes, which demonstrates that the use of multivalent vaccines in elderly individuals promotes robust immune responses and protects against multiple disease-causing pathogens [71,72]. This phenomenon highlights the necessity for developing novel vaccination strategies that boost immunogenicity in the elderly population through higher antigen doses or booster shots.

Modulating immune function is significantly dependent on the gut microbiome [73]. Poor health outcomes have been associated with changes in microbiomes, such as a decrease in microbiota diversity. Moreover, the pathophysiology of autoimmune diseases is greatly influenced by the gut microbiota [74]. Different bacterial communities were discovered through various mouse models. Gut microbiota dysbiosis leads to increased colonic oxidative stress, permeability alterations, and inflammatory reactions, interfering with barrier function and resulting in tissue damage and elevated markers of autoimmune disease. Bacteroides fragilis polarizes macrophages to an M1 phenotype, which improves their phagocytic capabilities [75]. According to the study by Schulthess et al., butyrate a short-chain fatty acid (a metabolite produced by bacteria), differentiates macrophages with stronger antimicrobial activity that produce more antimicrobial peptides [76]. Compared to the younger group, the elderly group had lower levels of Bifidobacterium and Lactobacillus [77]. Elderly subjects who consumed Bifidobacterium lactis HN019 for three weeks had a high number of natural killer cells, Th cells, activated T cells, and total T lymphocytes [78]. Additionally, the probiotic supplementation increased the natural killer cells’ ability to kill tumors and the ex vivo phagocytic ability of polymorphonuclear and mononuclear phagocytes. By supplementing Bacillus subtilis CU1, elderly subjects experienced a notable decline in the frequency of respiratory infections and a rise in IgA levels in their saliva and feces [79]. Although the mean number of days with common infectious disease symptoms did not decrease statistically, this result does point to the possibility that Bacillus subtilis CU1 may be a safe and efficient method of improving immune responses in the older population. The Mediterranean diet has been associated with favorable gut microbiota traits, such as elevated levels of Candida albicans, a higher ratio of *Bifidobacteria* to *Escherichia coli*, and lower counts of *Escherichia coli* [80]. Reintroducing *Lactobacillus plantarum* into the gut of aged mice can restore dendritic cell tolerance, which is diminished due to aging-associated gut microbiota dysbiosis. By supporting a balanced gut microbiota and optimal gastrointestinal function, this diet may be a helpful strategy for managing immunosenescence.

A study by Mondal et al. reveals that Δ133p53 and p53β play a role in cellular growth and aging, potentially opening a new therapeutic avenue for immunosenescence disorders [42]. Future research should focus on targeting p53β and Δ133p53 treatment, maintaining optimal p53 isoform balance, and exploring therapeutic interventions to modulate expression. Long-lived B cell depletion may aid in immunological competence enhancement and B-lineage rejuvenation, indicating that in the B-lineage, immunosenescence is not permanent [81]. There is evidence to support the idea that medications like metformin may reduce oxidative stress and enhance mitochondrial function, two things that are essential for preserving the health of immune cells in the elderly [82].

Regular physical activity can improve immune responses in the elderly population. In contrast to sedentary women, those engaging in physical activities have increased NK and T cell function, according to a study by Nieman et al. [83]. A study by Bartlett et al. suggests that decreased physical activity in older adults may contribute to impaired neutrophil migration, while regular physical activity may be beneficial for neutrophil-mediated immunity [84]. One approach to combat the age-related decrease in cellular immune response is to take vitamin E supplements [85]. Elderly participants, especially those with lower baseline immune responsiveness or less physical activity, showed improvements in delayed-type hypersensitivity and interleukin-2 production after taking a 100 mg vitamin E supplement for six months. These comprehensive approaches involving molecular, lifestyle, and gut health changes could enhance the immune response in vulnerable populations.

## 5. Conclusions

**Figure 4:**
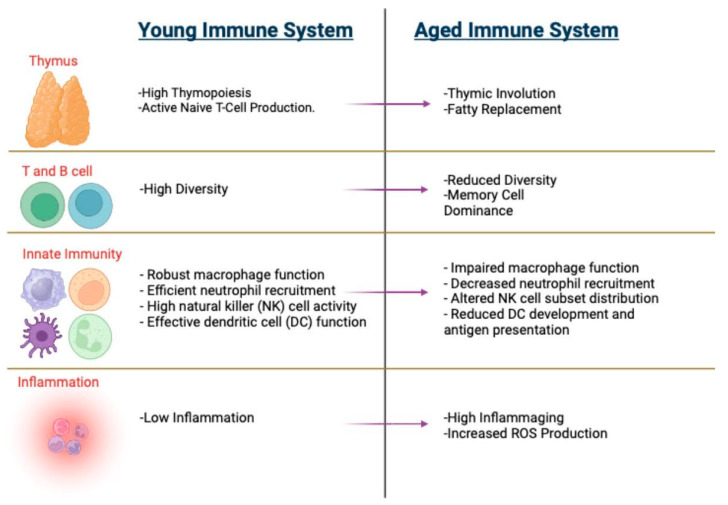
Comparative overview of young and aged immune systems. Key differences between young and aged immune systems. Young immune systems feature high thymopoiesis, naive T cell production, diverse T and B cells, and robust innate immunity. In contrast, aged immune systems exhibit thymic involution, memory cell dominance, impaired innate immunity, and increased inflammation.

Immunosenescence negatively affects the health and well-being of older populations by affecting various immune cells (see Figure 4 for comparison of young vs. aged immune systems). The complex interplay between genetic, molecular, and cellular processes plays a crucial role in the aging immune system. Key phenomena such as thymic involution, inflammaging, alterations in metabolic processes, and hematopoiesis underscore the multifaceted nature of immune decline. These changes result in diminished immune surveillance, reduced vaccine efficacy, and heightened vulnerability to infections and age-related diseases, such as cardiovascular diseases, autoimmune diseases, neurodegenerative diseases, cancers, and COVID-19. Comprehensive approaches, including dietary supplementation, lifestyle interventions, pharmacological treatments, and physical activity, may help to manage age-related immune decline and promote healthy aging. By combining these approaches, it may be possible to develop effective strategies for enhancing gastrointestinal health, promoting a balanced gut microbiota, and improving immune responses in older adults.

Investigating the molecular causes of immunosenescence, such as the functions of p53 isoform control, telomere attrition, and mitochondrial malfunction, ought to be part of the goals of future studies. It is crucial to develop treatments that improve vaccine responses and restore immune cell function, particularly in older persons. Advancing our understanding of immunosenescence with continued research into clinical practice is vital. Systematically developing and implementing strategies to enhance vaccination responses and immunological function will undoubtedly improve the overall quality of life and health of the aging population.

## Data Availability

The data used in this paper was obtained from PubMed.
